# Good Laboratory Practices and Safety Assessments

**DOI:** 10.1289/ehp.0900884

**Published:** 2009-11

**Authors:** Richard A. Becker, Erik R. Janus, Russell D. White, Francis H. Kruszewski, Robert E. Brackett

**Affiliations:** American Chemistry Council, Arlington, Virginia, E-mail: rick_becker@americanchemistry.com; Crop Life America, Washington, DC; American Petroleum Institute, Washington, DC; Soap and Detergent Association, Washington, DC; Grocery Manufacturers Association, Washington, DC

Having confidence in scientific procedures and data is the *sine qua non* for determining the safety of chemicals and chemical products. For decisions of safety, there must be rigorous and thorough application of fundamental scientific practices, irrespective of the purpose of the study and where it is conducted—academic, industry, or a contract laboratory.

Investigations must be designed and conducted by experts; whenever possible, standardized and validated test methods and test systems should be used, test devices and instruments must be appropriately calibrated and their accuracy assured, and, most important, all of the data, including raw laboratory records, should be available for independent review. Good Laboratory Practice (GLP) requirements, based on these fundamental scientific principles and practices, are indispensable for providing scientific confidence in studies conducted for chemical safety determinations. These reasons explain why government agencies worldwide require GLP compliance, and why it is entirely appropriate for greater weight to be given to GLP studies than non-GLP studies that are only available as articles in scientific journals. In their commentary Myers et al. (2009) argued that noncompliance with GLP should not be used as the sole criterion for excluding studies from consideration in regulatory decision-making. We agree that GLP should not be the sole criterion, but we strenuously disagree with the authors’ mischaracterization of the purpose and function of GLP and with their conclusion that GLP has no utility for weighting the reliability of studies.

Evaluating the safety of any substance should include review of all relevant studies utilizing a systematic weight-of-evidence framework. Although not all studies that are useful for hazard characterization and risk assessment may be amenable to GLP (e.g., epidemiology and mechanistic studies, studies conducted before the acceptance of current GLP), this does not obviate their consideration. Each study, GLP and non-GLP, should be evaluated and weighed in accordance with fundamental scientific principles. Factors to be evaluated include *a*) verification of measurement methods and data; *b* ) control of experimental variables that could affect measurements; *c* ) corroboration among studies; *d*) power (both statistical and biological); *e*) universality of the effects in validated test systems using relevant animal strains and appropriate routes of exposure; *f*) biological plausibility of results; and *g*) uniformity among substances with similar attributes and effects. Regulatory agencies [Food and Drug Administration (FDA) and U. S. Environmental Protection Agency (EPA)] and the National Toxicology Program (NTP) require studies to be conducted in accordance with GLP (FDA 2005; NTP 2006; U.S. EPA 2007a, 2007b), and the Organisation for Economic Co-operation and Development (OECD) GLP principles (OECD 1998) apply to all OECD member countries.

Academic basic research is very different from regulatory research and testing. Academic research focuses on developing and evaluating new hypotheses, on creating novel methods, and on discovering new findings. Academic research is open to wide interpretation and may require significant additional studies to clarify and determine whether and how broadly the results apply. Although novel techniques and discoveries of academic investigations stimulate further research, they must also stand up to the scientific method: hypothesis formulation, hypothesis testing, and validation by independent replication. Independent replication provides critical information on the strength of the hypothesis and reliability of test methods. Inconsistent results can arise from use of novel techniques, different test systems, uncertainty and differences in test chemical composition and purity, and a myriad of other factors. These facts, in conjunction with the more limited availability of actual data in most journal publications, means regulatory agencies can face significant challenges in confirming the quality, performance, or data integrity of results obtained solely from information available from a typical article in peer-reviewed journals. Whereas all study records and data from GLP investigations are available to agencies, rarely, if ever, are such details made available as part of the peer-review process for publishing a manuscript in a scientific journal. This can limit the ability of an agency to independently evaluate conclusions or to conduct alternative analyses of the data. The challenges faced by the peer-review procedures of journals have been recently highlighted (Nature 2006), and it has been pointed out that “…scientists understand that peer review per se provides only a minimal assurance of quality, and that the public conception of peer review as a stamp of authentication is far from the truth” (Jennings 2006). Journal peer review relies on summarization of experimental procedures and results, and does not include examination of laboratory study records or raw data. The purpose for journal peer review is to judge whether the study has been conducted and reported according to internationally recognized, general scientific standards and whether the study meets the interest level for dissemination to scientific community. It is not designed to provide assurance of accuracy or to recalculate raw data, and it does not provide an opportunity for independent audit of the study. Myers et al. (2009) failed to clearly make these distinctions.

Relevant internationally agreed test methods are used by industry to generate toxicity data for safety determinations by regulatory agencies. Incorporation of GLP in these laboratory tests assures that written protocols and standard operating procedures for each study component are developed and carefully and completely followed. GLP also requires meticulous adherence to dosing techniques; the use of adequate group sizes to allow meaningful statistical analysis; characterization (identity, purity, concentration) of test and control substances, including dosing solutions; detailed recording of study measurements and data; and collection of all raw laboratory data in a manner that can be retained and made available for regulatory agencies to audit and reach independent conclusions. Quality control procedures, quality assurance reviews, and facility inspections are also used to monitor and enforce GLP compliance. The relevance, reliability, sensitivity, and specificity of most test methods required of industry by regulatory agencies are well understood because they have been subjected to extensive, round-robin validation programs conducted in numerous laboratories throughout the world. This high level of scientific rigor, in conjunction with the detailed processes of GLP, provides regulatory agencies increased confidence in both the relevance and quality of GLP scientific studies for safety decisions, and it is the reason it is wholly appropriate in regulatory decision making for greater weight and confidence to be afforded to studies conducted in accordance with GLP.
